# Management of atraumatic shoulder instability in physiotherapy (MASIP): a survey of physiotherapy practice

**DOI:** 10.1186/s12891-021-04677-9

**Published:** 2021-09-30

**Authors:** Caroline Coulthard, Mindy C Cairns, Deborah Williams, Ben Hughes, Anju Jaggi

**Affiliations:** 1 Surrey iMSK Service, Ashford and St. Peter’s Hospitals NHS Foundation Trust, Guildford Road, Chertsey, Surrey KT16 0PZ UK; 2 School of Health and Social Work, University of Hertfordshire, Hatfield, Hertfordshire AL10 9AB UK; 3 The Shoulder & Elbow Unit, Royal National Orthopaedic Hospital, Brockley Hill, Stanmore, HA7 4LP UK; 4 Wrightington, Wigan and Leigh NHS Foundation Trust, Wrightington Hospital, Hall Lane, Appley Bridge, Wigan, WN6 9EP UK

**Keywords:** Shoulder, Instability, Physiotherapy, Atraumatic, Survey, Glenohumeral, Management

## Abstract

**Background:**

The impact of atraumatic shoulder instability (ASI) on patients can be extensive, its management complex, with a biopsychosocial approach recommended. Currently how physiotherapists manage ASI is unknown or the extent to which current clinical practice aligns with existing evidence. At the time of this study no national guidelines or consensus to direct practice existed.

**Methods:**

A cross-sectional electronic survey was distributed between July-September 2018, targeting UK-based physiotherapists managing shoulder pathology.

Respondents were invited to describe their management of ASI, and rate their awareness and utilisation of various treatment techniques on a Likert-scale; median and interquartile ranges were calculated. Free text survey items were analysed using quantitative content analysis (QCA) to identify codes and categories. Means and percentages were calculated to summarise QCA and descriptive data.

**Results:**

Valid survey responses were analysed (*n* = 135). Respondents had between 2 and 39 years of physiotherapy experience (mean = 13.9 years); the majority (71.1 %) reported that ASI made up < 10 % of their caseload. Only 22.9 % (*n* = 31/135) of respondents reported feeling ‘very confident’ in managing ASI; the majority feeling ‘somewhat confident’ (70.4 %, *n* = 95/135) or ‘not confident’ (6.7 %, *n* = 9/135).

The majority of respondents (59.3 %) used an ASI classification system, > 90 % citing the Stanmore Classification. Physiotherapists adapted their management according to clinical presentation, responding to differing biopsychosocial needs of the patient scenario. Most respondents (> 80 %) did not use a protocol to guide their management. Exercise was the most utilised management approach for ASI, followed by education; novel treatment strategies, including cortical rehabilitation, were also reported.

**Conclusion:**

Findings indicate physiotherapists utilise a wide range of treatment strategies and respond to biopsychosocial cues when managing patients with ASI. The majority reported not being very confident in managing this condition, however only a minority use rehabilitation protocols to support their management. Some interventions that respondents reported using lacked evidence to support their use in ASI management and further research regarding effectiveness is required. Guidelines have been published since this survey; the impact of these will need evaluating to determine their effectiveness in the future.

**Supplementary Information:**

The online version contains supplementary material available at 10.1186/s12891-021-04677-9.

## Background

Shoulder pain is the third most common musculoskeletal complaint [1], constituting approximately 2.4 % of General Practitioners (GP) consultations [2], with an estimated 4 % suffering from recurrent shoulder instability in the absence of trauma [3] although there is no recent epidemiological data available. The impact of atraumatic shoulder instability (ASI) can be extensive, with excessive pain, psychological distress and functional impairment reported [4, 5]; a detrimental impact upon work and school attendance and high dependence upon healthcare resources [6].

Classification systems have been presented to facilitate clinicians in managing patients with shoulder dislocation; Thomas and Matsen highlighted differences between patient management depending upon whether shoulder instability was due to traumatic or atraumatic mechanisms [7]. The Stanmore Triangle classification makes further distinctions in patient management for ASI, allowing patients to be placed into one of three polar groups, or sub-classified between poles e.g. have a clinical history and or clinical presentations that fall between polar groups [8]. Patients with ASI can present with various associated pathologies including muscle patterning dysfunction, sensorimotor deficits, psychological conditions, central sensitisation, and hypermobility [9–13]. Consequently patients with ASI usually require a range of specialised assessment and treatment strategies to aid their management [6, 14]. Whilst physiotherapy is advocated, if this fails, surgery may be considered [15, 16].

No specific national guidelines or consensus opinion were available to direct physiotherapy management of ASI at the time of this study. Guidelines have been subsequently published by the British Elbow and Shoulder Society (BESS), based upon the best available evidence and expert consensus opinion [17]. These BESS guidelines recommend for initial physiotherapy management of ASI to include reassurance, education and suitable exercise prescription specifically targeting proprioception, and to include the muscles of the scapula and rotator cuff. Failure to improve after 12-weeks or in the presence of indicators for early referral; prompt onward referral is recommended. This may include imaging of the glenohumeral joint or specialist multi-disciplinary care where there are psychological and or social factors limiting engagement with physical exercise.

Management has historically been directed by expert opinion through clinical commentaries and consensus studies [13, 14, 16], rather than high quality evidence [17, 18]. Some authors suggest using classification systems to help guide clinical practice to help direct management [7, 8, 19, 20], which was echoed in a recent Delphi study of posterior shoulder instability [18].

Exercise-based rehabilitation is the primary approach recommended for ASI management [14, 16, 18, 21, 22]. Rehabilitation of the periscapular and rotator cuff muscles are widely recommended for ASI [14, 15, 18, 21, 23, 24] and are specifically cited in the recently published national guidelines for managing ASI [17]. Additionally proprioceptive exercises [9, 15, 16, 21, 23, 25], flexibility exercises [9, 18, 21] and functional and sport specific training [9, 16, 18] are frequently highlighted. The extent to which physiotherapists have adopted specific types of exercise, such as rotator cuff training, kinetic chain, or exercise-based protocols within their clinical practice when managing ASI is unknown.

The Watson Multidirectional Instability (MDI) Programme demonstrated positive functional improvements following a 12-week structured exercise-based programme, incorporating scapula posture correction, strengthening, functional and sports-based rehabilitation [26]. The Derby Shoulder Instability Rehabilitation Programme (DSIRP), which additionally incorporates proprioceptive and kinetic chain exercise, has also demonstrated beneficial short-term clinical outcomes [27, 28]. However, these are single-group studies carrying a high risk of bias. A randomised controlled trial further supports the clinical effectiveness of the Watson MDI Programme, when compared to the Rockwood Instability Programme [29], however a need for further high-quality evidence is required [22, 23].

Consideration has been given in the literature to how psychosocial factors and emotional wellbeing could influence rehabilitation and the potential for recovery for people with ASI [13, 30, 31]. It is recommended that signs of movement related fear and anxiety are addressed in the early stages of management through reassurance, education and early mobilisation [13, 14, 16, 30], which reflects the importance placed on these strategies within the recently published guidelines [16]. A small number of authors also recommend the provision of suitable education for patients in order to explain the role of rehabilitation, optimise pain management, promote understanding of ASI and the general anatomy and function of the shoulder [13, 18], as well as validation of the patient experience [31].

Other interventions to manage ASI include symptom modification, compression and functional electrical stimulation (FES) [13, 14, 31, 32], as well as emerging or novel approaches to ASI management such as Graded Motor Imagery (GMI) or neurodevelopmental training [13, 31, 33]; however clinical effectiveness of these approaches in ASI has not be established.

Recent evaluations of clinical practice for managing other upper limb disorders have demonstrated clinical variation and non-evidence based practice [34–36]. To date, little is known regarding the management of ASI among physiotherapists. The aim of this study was to establish current physiotherapy management of patients with ASI in the United Kingdom (UK).

The objectives of the research were to:

- Establish current clinical practice for the management of ASI.
- Identify the extent to which certain ‘novel’ treatment strategies are used for managing ASI.

## Methods

The survey design was used based on a similar methodology in previous surveys of physiotherapy practice [34–38], based on the available evidence and in conjunction with feedback from The Shoulder & Elbow Unit Team at The Royal National Orthopaedic Hospital, Stanmore. The Checklist for Reporting Results of Internet E-Surveys (CHERRIES) was used to guide reporting [39].

### Survey content

The survey comprised of twenty-eight questions. Demographic questions relating to level of experience and clinical practice were included to establish baseline information regarding the sample population. To evaluate clinical practice, a 5-point Likert-scale for respondents to rate their awareness and usage of a range of management strategies were utilised, and three clinical vignettes describing different patient presentations based upon the Stanmore Classification System [8] with a combination of open and closed questions (see Additional file 1 for full survey).

The Stanmore Classification acknowledges that in shoulder instability there is not always a single identifiable cause or pathology and that multiple pathologies may co-exist. It also acknowledges that there is a continuum between the different pathological groups. Type I (traumatic structural), Type II (atraumatic structural), Type III (non-structural, neurological or abnormal muscle patterning). which are diagrammatically represented on the Stanmore Triangle [16]. Vignette 1 (V1) was designed to reflect a patient fitting Type II on the Stanmore Triangle, Vignette 2 (V2) and Vignette 3 (V3) had more complex ASI presentations, being designed to reflect Type III and Type III(II) respectively (See Table 1).

**Table 1:** Vignette descriptions

**Vignette 1 *(Type II instability)*** Twenty-six-year-old male with atraumatic shoulder instability. Works as an accountant and used to play county cricket but stopped due to developing difficulty with throwing. He has avoided excessive overhead use since then. Complains of sensation of instability and discomfort in right shoulder, particularly on reaching overhead and gardening; been going on for a few years. He had a Magnetic Resonance Arthrogram (MRA) which shows Bankart lesion of the anterior labrum. He has been referred to physiotherapy by an Extended Scope Practitioner. He now wants to get back to fitness and has started running and upper body weight training. He is struggling with weights due to pain. He wonders whether exercise is the right thing for his shoulder. On examination he has full range of movement, with notably excessive external rotation. He has a positive gleno-humeral internal rotation deficit (GIRD) test. He has positive anterior apprehension test.
**Vignette 2 *(Type III instability)*** Twenty-four-year-old female with bilateral shoulder pain, sensation of instability and history of multiple atraumatic shoulder dislocations. Sometimes attends A&E for help with relocation but normally able to self-relocate, but shoulder will often pop out again shortly after. Owns a dog but struggles to control it on-lead due to feeling shoulder will come out. Unable to work for the past 6 months due to symptoms. Started volunteering at a dog rescue centre but can’t walk the dogs due to shoulder. Has had episodes of physiotherapy previously, including hydrotherapy and strengthening – feels this hasn’t helped overall. Seen a consultant and told not a surgical candidate as no structural pathology on imaging and referred back for another go at physio. Patient is worried that it won’t help again.
**Vignette 3 *(Type II - III instability)*** Twenty-six-year-old female office worker. History of shoulder bilateral atraumatic shoulder instability with multiple subluxations. Has good social support but lives a relatively sedentary lifestyle. Told was hypermobile when she was younger. No past medical history. Avoids lifting and reaching overhead due to feeling that shoulders will come out. On examination she has reduced active range of shoulder elevation due to pain, and reports feeling unstable. Range of movement is full passively, with 90 degrees external rotation. Her Beighton score is 7/9. She has a positive sulcus sign, anterior and posterior load and shift, and anterior apprehension and relocation test.

Additional questions were used to explore clinicians’ views regarding future research and training. The clinical vignettes were based upon the varying presentations of ASI [9], and are a validated method of evaluating clinical practice among physiotherapists [40].

### Survey validation

For validation, expert-driven pre-testing was undertaken to review preliminary survey content before adjustments were made to demographical and ASI management questions. The survey was subsequently piloted in its online format using practicing physiotherapists with a range of musculoskeletal experience and interest in shoulder instability. Feedback regarding content and format was received via individual respondent debriefing sessions, which led to minor modifications to the vignettes to encourage more detailed answers regarding physiotherapy management. A survey draft was distributed informally to an upper limb professional physiotherapy network available to the author. Respondents were asked to review and feedback in relation to the content, format and general suitability of the survey based upon the project aims. Final modifications were made to the vignettes to include more potential shoulder instability classification systems, make clearer distinctions between further management options, and expand the range of treatment strategies for participant self-evaluation.

### Sample

Based upon the distribution plan for the survey, and response rates from previous comparable surveys of UK-based physiotherapists [34, 36, 37, 41], an overall response rate of 100–250 was anticipated.

### Inclusion and exclusion criteria

Respondents had to be Health & Care Professions Council (HCPC) registered Chartered Physiotherapists practising in the UK. This was confirmed by a filter question at the beginning of the survey. The survey marketing was targeted towards physiotherapists with experience of managing patients with shoulder pathology.

### Ethical considerations

 Implied consent was obtained through participants having to opt into the study. A participant information sheet was included at the beginning of the survey. A survey completion receipt and a copy of the participant’s responses were made available to them to facilitate learning and reflection.

 A favourable ethical opinion was obtained from University of Hertfordshire Health, Science, Engineering and Technology Ethics Committee with Delegated Authority (HSK/PGT/UH/03371). The project was approved by Ashford & St Peter’s Hospitals NHS Foundation Trust Office of Research & Development (Ref. 2018CC01SP).

### Distribution

The questionnaire was hosted online by Online Surveys (www.onlinesurveys.ac.uk). A link to the survey was advertised by the Chartered Society of Physiotherapy (CSP) to their members through Frontline magazine and the interactive online message-board (https://www.csp.org.uk/icsp). The Musculoskeletal Association of Chartered Physiotherapists (MACP) independently reviewed and approved the survey for distribution via their own social media network (882 members), and permission was granted from the British Elbow & Shoulder Society (BESS) to publicise the survey at their 2018 Glasgow conference through a PowerPoint slide and business cards to 101 attendees.

The survey was also circulated via social media (Twitter, LinkedIn, Facebook) and to relevant professional contacts of the author. The survey opened on 4th July 2018 and remained active for 2 months.

### Analysis

Descriptive analysis was used to describe the sample population, and to summarise level of confidence in managing ASI, the reported use of classification systems, and the reported use of rehabilitation protocols. Median and interquartile range were calculated to summarise Likert-scale data on awareness and utilisation of a selection of ASI management strategies. All analyses were carried out using Microsoft Excel (2010).

Free text survey items were analysed using Quantitative Content Analysis (QCA) [42] to identify codes and categories. Seven categories were generated in advance of analysis (deductively), with these being education, Psychologically Informed Therapy (PIT), onward referral, alternative management strategies, cortical rehabilitation, exercise, manual therapy, and a further seven were generated deductively during the analysis (exercise approach, management aim, assessment, outcome measures, respondent perspective, psychosocial, clinical reasoning) [43]. A second researcher undertook reliability checking of the coding process, using randomly selected extracts of data [44]. The primary focus of QCA was to identify the range of treatment strategies utilised to manage ASI, and the extent to which individual treatment strategies are utilised. The secondary focus was to recognise whether, and how, respondents adapted their management between the three clinical vignettes. Relative frequency percentages were calculated to summarise the QCA data.

## Results

### Demographical sample population data

A total of 149 responses were received; of those 13 were ineligible to participate as they were not based in the UK, and one failed to complete the survey correctly. Therefore, a total of 135 responses were analysed, and Table 2 summarises the descriptive data of the respondents. Respondents had been practising physiotherapy for a mean of 13.9 (S.D. 8.0) years and 48.9 % had over 10 years of musculoskeletal experience. The majority of respondents were in senior roles, with 68.8 % working at or above NHS Band 7 level (or equivalent). Most respondents (77.8 %) worked in the National Health Service (NHS) as their primary role.

**Table 2:** Descriptive sample data

Overall physiotherapy experience (years)	Mean = 13.9 (S.D 8.0)	
**Band / Grading**		
Band 5 *(Physiotherapist)*	4	*3.0 %*
Band 6 *(Physiotherapist Specialist)*	38	*28.1 %*
Band 7 *(Physiotherapist Advanced / Specialist Physiotherapist / Physiotherapy Team Manager)*	47	*34.8 %*
Band 8a *(Physiotherapist Principal)*	40	*29.6 %*
Band 8b upwards *(Physiotherapist Consultant)*	6	*4.4 %*
**Setting**		
NHS	105	*77.8 %*
* Primary*	*37*	
* Secondary*	*45*	
* Tertiary*	*6*	
* Mixed*	*17*	
Private hospital	10	*7.4 %*
Private practice	18	*13.3 %*
Education	1	*0.7 %*
Sports	1	*0.7 %*
**Overall MSK experience (years)**		
0–2	8	*5.9 %*
> 2–4	9	*6.7 %*
> 4–6	19	*14.1 %*
> 6–8	18	*13.3 %*
> 8–10	15	*11.1 %*
> 10	66	*48.9 %*
**Upper Limb Specialism**		
Yes	68	*50.4 %*
* Interest group organisation*	*32*	
* Clinical / special interest in ASI*	*58*	
* Work in specialist ASI centre*	*24*	
No	67	*49.6 %*
**ASI Training**		
Yes	115	*85.2 %*
No	20	*14.8 %*
**ASI Experience Outside Workplace**		
Yes	19	*14.1 %*
No	116	*85.9 %*
**Proportion of patients with ASI on caseload**		
None	3	*2.2 %*
> 0–10 %	93	*68.9 %*
> 10–30 %	25	*18.5 %*
> 30–50 %	5	*3.7 %*
> 50–75 %	5	*3.7 %*
> 75–100 %	4	*3.0 %*

Approximately half (50.4 %) of respondents were upper limb specialists; however ASI comprised less than 10 % of the clinical caseload for the majority (71.1 %) of respondents. Less than a quarter of respondents (22.9 %) reported feeling ‘very confident’ in managing ASI.

### Vignette responses

**Fig. 1:**
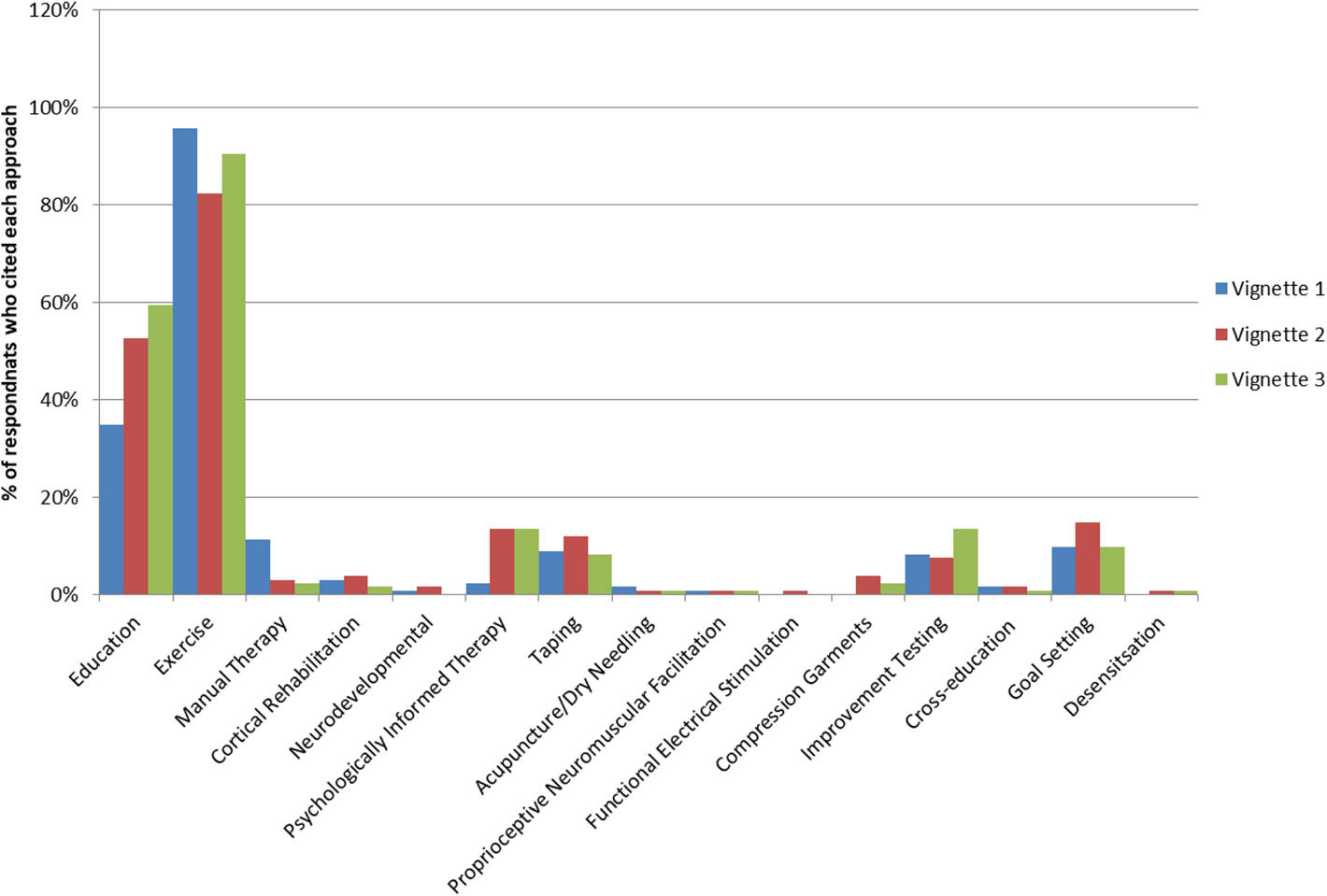
Physiotherapy management modalities identified according to vignette

Of the respondents, 59.3 % chose to use an ASI classification system in the vignettes. Most of those respondents (92.5 %) referenced The Stanmore Classification in at least one vignette. A wide range of management options were utilised for the three different vignettes; Fig. 1 illustrates the proportion of respondents that selected different treatment modalities. The most popular treatment modality across all vignettes was exercise (V1 95.6 %; V2 82.2 %; V3 90.4 %), followed by education (V1 34.8 %; V2 52.6 %; V3 59.3 %).

**Fig. 2:**
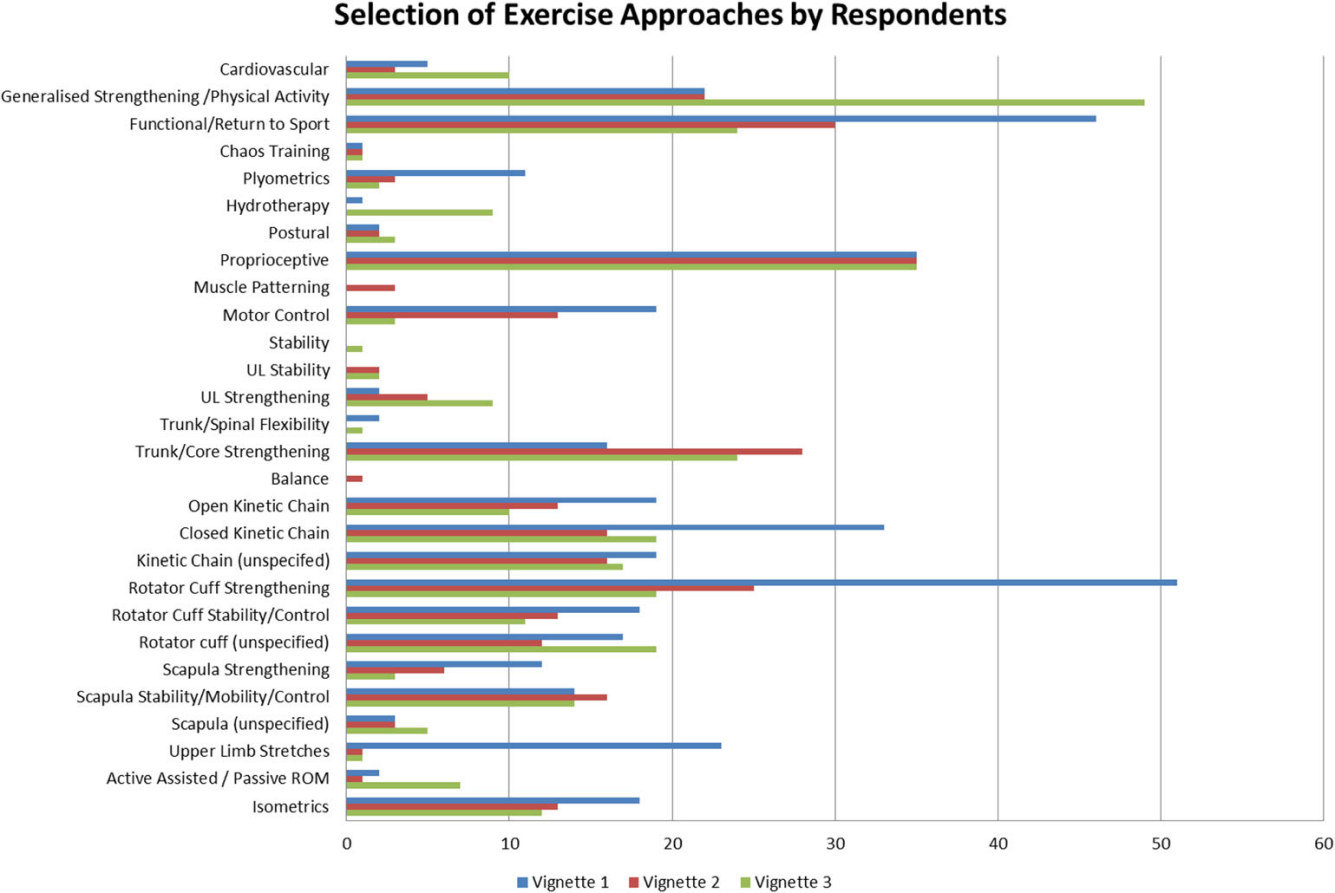
Exercise strategies reported according to vignette

The types of exercise selected by respondents varied according to the vignette (Fig. 2). Isolated rotator cuff strengthening (39.5 %) and closed kinetic chain exercises (25.6 %) were cited more frequently for V1, whereas general strengthening and physical activity (40.2 %) were cited more often for V3. Core and trunk strengthening featured more commonly for V2 (25.2 %) and V3 (19.7 %).

**Fig. 3:**
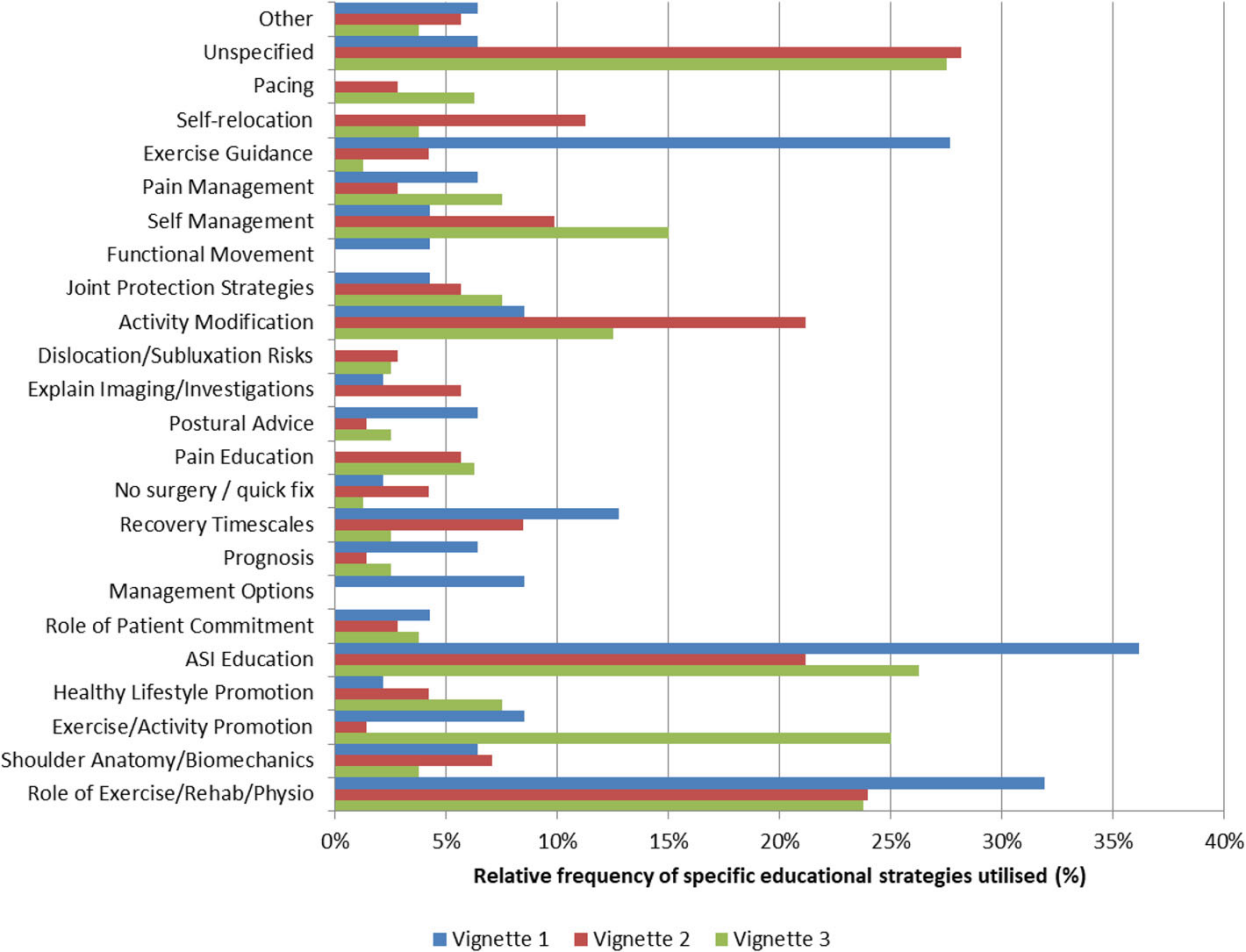
Education reported according to vignette

Of those respondents who cited education within their management, the specific forms of education described are shown in Fig. 3. Respondents reported ASI (including hypermobility) education (V1 36.2 %; V2 21.1 %; V3 26.3 %) and explanation of the role of physiotherapy (V1 31.9 %; V2 23.9 %; V3 23.8 %) for all three vignettes. Types of education varied between vignettes, with exercise guidance cited more frequently for V1 (27.7 %), activity/exercise promotion cited more frequently for V3 (25.0 %) and activity modification for V2 (21.1 %).

A minority of respondents (18.5 %, *n* = 25/135) identified using a specific protocol to manage ASI, however only 64.0 % (*n* = 16/25) of these were using a recognised evidence based ASI protocol; the majority (93.8 %, *n* = 15/16) of these referenced the DSIRP in at least one vignette.

### Management of psychosocial factors

The use of Psychologically Informed Therapies (PIT) were cited by 13.3 % (*n* = 18/135) of respondents for both V2 and V3 (Fig. 1). The most popular PITs in V2 being functional restoration (5.2 %; *n* = 7/135) and cognitive behavioural therapy (4.4 %; *n* = 6/135).Graded exposure was the most popular strategy for V3 (5.2 %; *n* = 7/135). In contrast, only 2.2 % (*n* = 3/135) of participants cited the use of PIT for V1. Respondents cited the importance of addressing psychosocial factors as aims of management, with reassurance being the most reported consideration across all vignettes (V1 14.8 %, V2 19.3 %, V3 17.0 %). In addition, optimising patient engagement was frequently reported for V2 (8.9 %), and building confidence (11.1 %) and addressing patients fears (15.6 %) were also reported for V3.

### Onward referral if physiotherapy was unsuccessful

**Fig. 4:**
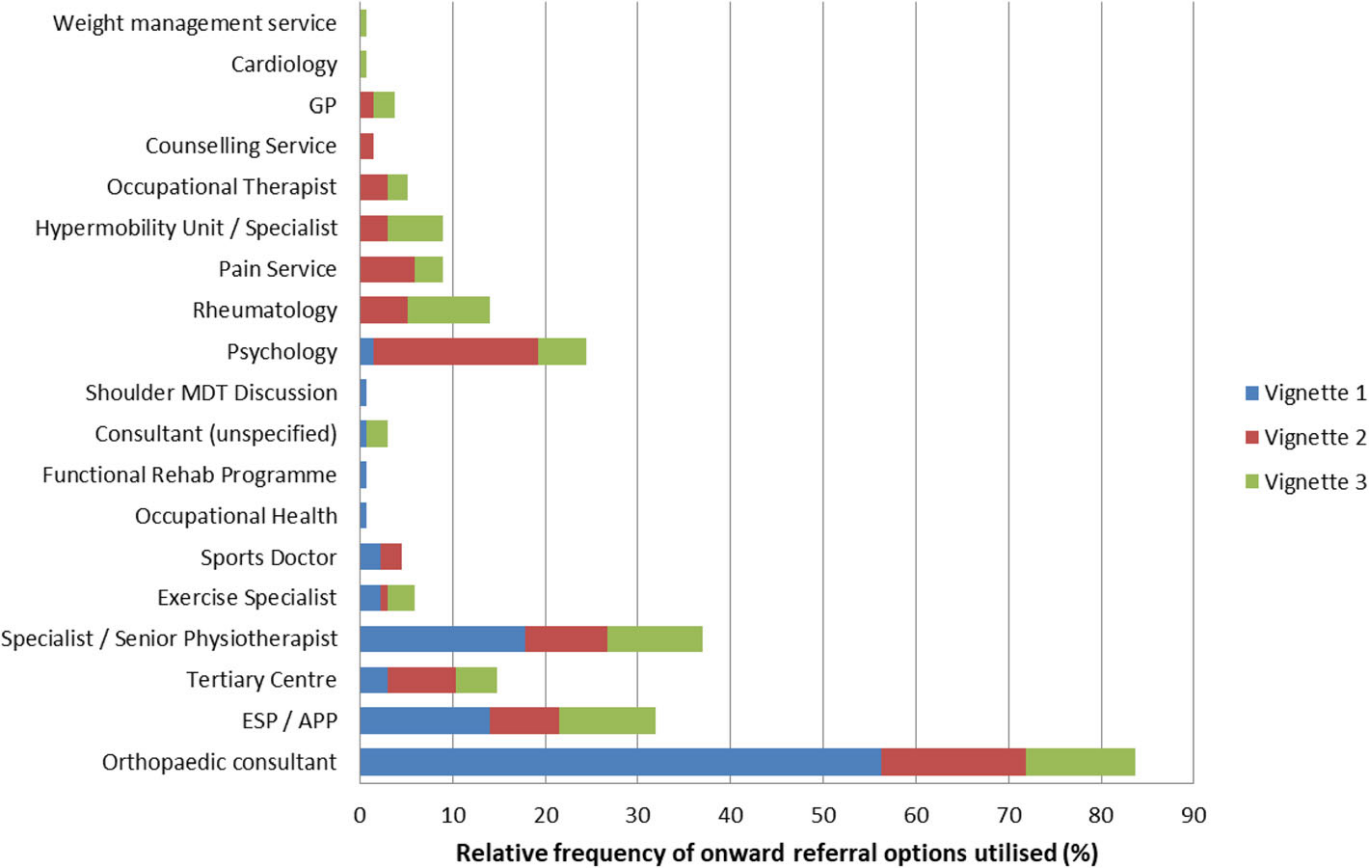
Physiotherapy onward referral strategies according to vignette

In response to the question regarding onward referral if physiotherapy was unsuccessful, a range of referral options were cited.Onward referral was most frequently reported for V1 (83.7 %), however fewer respondents reported onward referral for V2 (58.5 %) and V3 (55.6 %). For V2 and V3, a wider range of onward referral options were cited by respondents, and these are displayed in Fig. 4.

The types of onward referrals reported by respondents varied between the vignettes. Referral to an orthopaedic consultant (56.3 %) was the most frequently cited option for V1; the most frequent reasons given for onward referral for V1 were for surgical opinion (41.5 %), with only 12.6 and 11.1 % of respondents wanting specialist opinion or second opinion for physiotherapy management respectively. Referral to psychology (17.8 %) was the most frequently reported for V2, with the most frequent reasons being to address psychological or psychosocial factors (12.6 %), with 15.6 % still citing referral to an orthopaedic consultant and 9.6 % wanting a surgical opinion. Referral to an orthopaedic consultant (11.9 %), an ESP/APP (10.4 %), or specialist physiotherapist (10.4 %) were most commonly cited for V3, however, the reasons for onward referral were for specialist opinion (6.7 %) and for addressing rheumatological factors (7.4 %).

### Respondent self-reported awareness and usage of treatment strategies

**Fig. 5:**
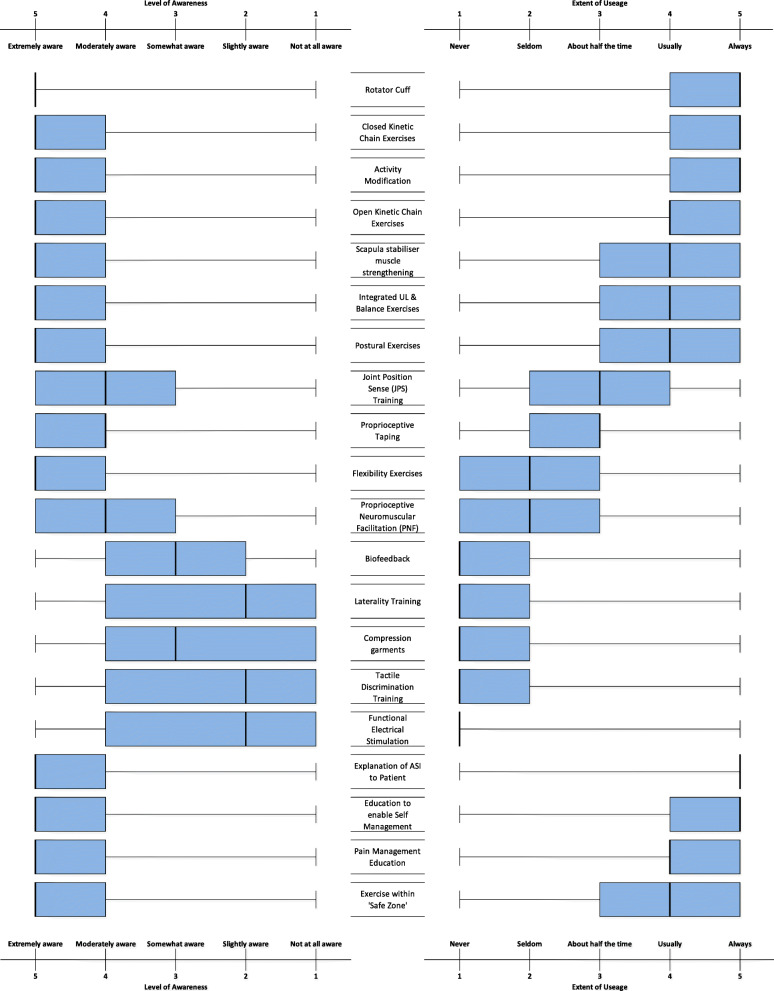
Box plot demonstrating awareness and usage of ASI

Figure 5 displays mean scores of respondent self-reported level of awareness and usage of specific ASI treatment strategies that have been recommended by experts [13, 14, 16, 21, 31]. Respondents rated themselves as ‘extremely aware’ of a range of different exercise approaches for managing ASI including open and closed kinetic chain exercises, with rotator cuff exercises having the highest agreement among respondents. There appeared to be a high correlation between awareness and extent of usage for exercise and educational strategies overall.

The least reported management strategies, which may be considered as novel treatment approaches, were use of compression garments, tactile discrimination, functional electrical stimulation, biofeedback, and laterality training. These ‘novel’ treatment approaches showed much larger disparity between level of awareness and extent of usage, with respondents being much more aware of these approaches, compared with the extent to which they used them.

### Future ASI training and research

Clinicians were asked what research priorities should be in this area. Clinical guidance and effectiveness of management strategies were both cited most commonly by 19 % (*n* = 25/135). Additionally, respondents were asked what types of training they would like to receive regarding ASI management with participants favouring a wide range of training options, therefore further investigation in this area may be beneficial.

## Discussion

### Main findings

This is the first study to investigate how physiotherapists manage ASI in the UK.

Exercise was the most frequently cited intervention, followed by education. Respondents reported adopting a biopsychosocial approach in managing patients with ASI and varied the intervention depending upon clinical presentation and psychosocial needs of the patient.

### Confidence in managing ASI

Less than a quarter of physiotherapists reported themselves as being ‘very confident’ in managing ASI, which was unexpected in a sample where 50.4 % self-identified as shoulder specialists. This lack of confidence could reflect the disparity of strong evidence available to support specific management strategies for ASI, or reflect the limited exposure to ASI in their clinical practice. The majority of physiotherapists (71.1 %) reported that these patients make up less than 10 % of their clinical caseload, consistent with the low prevalence of ASI estimated in the literature [3].

### Use of classification systems

Almost 60 % of physiotherapists reported utilising a classification system for ASI, with almost all of those (92.5 %) using the Stanmore Classification system in at least one vignette. A key advantage of this system is its flexibility; patients can be categorised at one of three poles of a triangle, in which case they will exhibit a defined set of clinical features, or on a continuum between poles, as is often the case [45]. Patients with ASI are considered complex to manage [9, 46] and the Stanmore system is designed to encompass the range of clinical presentations, including muscle patterning disorder, structural deficit, and hypermobility, to support clinicians in stratifying their management approaches accordingly. However, reliability has been questioned, attributable in part to the complexity of clinical presentation [4, 16, 47]. Given the low confidence in managing ASI reported in the present study and the popularity of the Stanmore system, findings of this study indicate that physiotherapists may benefit from guidance published after this survey [17].

### Exercise

Exercise was the most frequently cited treatment intervention reported by respondents for managing the clinical vignettes, which is supported by expert opinion and the limited research available supporting non-surgical management of ASI [27, 30, 48–50]. Interestingly, physiotherapists reported adapting their exercise approach depending upon the clinical scenario, aligning with expert recommendations [9, 13] but in conflict with the evidence supporting the clinical effectiveness of standardised rehabilitation protocols [27–29, 48].

In adapting their exercise-based management, physiotherapists cited a range of specific exercise types including rotator cuff, scapular strengthening, functional and return to sport exercises more frequently for V1 than the other vignettes. These exercises correlate well with the recommended exercises within the BESS guidelines [17], and additionally correlate closely with those incorporated within exercise-based protocols. This indicates that for V1, designed to reflect a patient fitting Type II on the Stanmore Triangle, exercise-based protocols may be considered most suited to this patient group amongst respondents of the present study. Vignette 2 and 3 had more complex ASI presentations, being designed to reflect Type III and Type III(II) on the Stanmore Triangle respectively. Generalised exercise approaches, including trunk strengthening and physical activity, were cited more frequently for these vignettes. These types of exercises have been recommended as part of stratified management for patients of Types II and III on the Stanmore Triangle, but are not recommended at the cost of more specific rehabilitation of the rotator cuff and peri-scapular muscles [9, 17]. Findings in the present study reflect variability in exercises prescribed for patients with ASI, with more complex patients potentially receiving more generic exercise programs and appearing to be less likely to receive exercises specifically recommended within the BESS guidelines, which itself could impact the success of exercise-based management and deliver sub-optimal outcomes.

On reviewing the literature; there is a paucity of high-quality evidence to guide physiotherapists in prescribing specific forms exercise when managing patients with ASI, and further research is required. Physical activity can be prescribed using the FITT (Frequency, Intensity, Timing, Types) Principle [51–53], and high-quality research incorporating this principle, may be beneficial in establishing the optimal exercise approach for managing patients with ASI.

### Exercise-based protocols

It was expected that physiotherapists might favour protocols to guide their management of this complex group and tailor aspects of management in conjunction with protocol usage, particularly given the low confidence in managing ASI reported from respondents. However, only 18.5 % of respondents reported using an exercise-based protocol for their management. This low uptake could reflect a lack of awareness of protocols among the profession, which was not anticipated considering the level of shoulder specialism within the sample population, or could indicate that protocols are not entirely meeting the needs of patients with ASI, or the physiotherapists supporting them.

### Education & advice

Education was the second most reported management strategy. Vignettes were deliberately designed to reflect different psychosocial needs, and respondents appeared to tailor the types of education to each vignette. Education was unspecified more frequently by respondents for V2 and V3, which could reflect the complexity of these patients and indicate a knowledge gap among respondents. Education featured within vignette management almost 50 % less than exercise, despite physiotherapists reporting that they were equally as aware of each of these approaches. This may reflect the focus of research and clinical commentaries on exercise-based management in comparison to educational strategies [9, 13, 16]. In the wider research available on shoulder pain, there is emerging evidence in support of tailoring education to the patient, with activity and general exercise promotion, for example, recommended in patients who have a sedentary lifestyle [54]. Education is advocated in managing ASI following a recent case series involving 85 patients [55] and also features in the BESS guidelines [17]. Given the high utilisation of education, there is justification for further research to investigate this aspect of management and inform future practice.

### Psychologically Informed Therapies

Psychologically Informed Therapies (PIT) were cited for V2 and V3 more frequently than they were for V1, which may reflect the greater number of psychosocial factors that these scenarios included. This was further reflected in where respondents would refer patients elsewhere, should they not respond to physiotherapy management. Findings indicate that physiotherapists are both identifying, and determining that there is a need to manage psychosocial factors, which is in line with evidence highlighting the psychological impact of ASI on the patient [5]. One of the most popular aspects of managing psychosocial factors was reassurance, which is recommended in the BESS guidelines [17]. To date, there is a paucity of research supporting the clinical effectiveness of PIT and addressing psychosocial factors in managing ASI, and only limited evidence available to support their role in shoulder pain [54]. As psychosocial drivers are thought to have an impact on ASI [13, 14, 30, 31], physiotherapists may be extrapolating evidence from other conditions to support their utilisation of both education and PIT in order to address patient needs [56–58].

### Novel management approaches

Physiotherapists cited a higher awareness of novel treatment approaches for managing patients with ASI, compared to the extent to which they reported utilising them. This discrepancy could reflect a lack of confidence in utilising these more novel treatment approaches or reflect the paucity of evidence for clinical effectiveness in treating patients with ASI. Some of these approaches have evidence to support their efficacy in ASI, such as compression garments in improving proprioception [59, 60]. Conversely, some strategies such as cross-education, cortical rehabilitation, and functional electrical stimulation, have no specific evidence for clinical effectiveness in ASI, and evidence can therefore only be extrapolated from research into other conditions [61–63]. Interestingly, physiotherapists cited a higher awareness of novel treatment approaches for managing patients with ASI, compared to the extent to which they reported utilising them, which was observed for popular strategies such as exercise and activity modification. This discrepancy could reflect a lack of confidence in utilising these more novel treatment approaches, or reflect the paucity of evidence for clinical effectiveness in treating patients with ASI.

### Study strengths and limitations

The study achieved a reasonable number of completed responses in comparison to other upper limb clinical practice surveys, suggesting that distribution strategies were successful. Due to the distribution methods however, it was not possible to identify a specific sample or record how many potential respondents had access to the survey, therefore an accurate response rate could not be calculated. Comparison with national data suggest that the sample was broadly demographically representative of UK-based physiotherapists [64]. However, there was an over-representation of physiotherapists working at NHS Band 8 or equivalent, and an under-representation of those working at NHS Band 5 or equivalent, indicating selection bias.

The use of vignettes is well established method of gaining information regarding current clinical practice [40] but other methodologies such as focus groups [65] or face to face interviews [66] might have allowed a greater investigation of specific issues related to management.

This seniority within the sample could explain why 50.4 % of respondents reported a shoulder specialism, and may bias the findings in favour of clinicians who are more informed regarding current research evidence. Conversely, this population may be more reflective of the population who manage this complex patient group, with a number of patients being managed within tertiary care centres having contact with specialist physiotherapists.

### Implications for clinical practice and future research

This study highlights variation amongst physiotherapists in how they manage patients with ASI. There was a tendency for approaches that are most widely recommended in the literature and within the BESS guidelines to be most commonly reported to be used by physiotherapists in the present study. However a number of physiotherapists were not delivering some of these recommendations at all or not delivering them consistently. Dissemination of evidence-based guidelines for clinicians working with patients with ASI at primary care level is recommended in order to build a solid foundation for clinical practice. The use of widely accessible media such as podcasts and infographics may provide an initial platform for sharing this information amongst those physiotherapists managing patients with shoulder pain. For those physiotherapists working within secondary care, supporting the development of management pathways to complement the BESS guidelines may minimise clinical variation, and promote the onward referral as appropriate. Specific guidance on how to structure and deliver the expanded assessment and management approaches such as sensorimotor integration and the management of central sensitisation may be best delivered through specialist clinical commentary or more detailed physiotherapy-specific guidance. In addition, this group often present with co-morbidities such as joint hypermobility spectrum disorder and or chronic persistent pain states that may be more related to these aspects than specifically to the shoulder.

Education and PIT were reported widely amongst respondents and are supported by evidence highlighting the psychological and functional impact of ASI on patients [4–6]. Qualitative exploration of psychosocial factors affecting patients with ASI is recommended to help direct future education and psychology-based management, and to produce evidence-based recommendations to support clinical guidance and optimise patient care.

Exercise is the most utilised approach reported, however there was variation in types of exercise selected for the differing scenarios and some patients with ASI would be less likely to receive recommended exercise approaches compared to others. There is a paucity of high-quality research to guide the clinician in exercise prescription. Exercise-based management protocols have the potential to offer guidance to physiotherapists who lack confidence in managing ASI, however with limited availability of high-quality evidence; further research is recommended to demonstrate their clinical effectiveness. The lack of robust evidence may be a barrier to physiotherapists using such protocols, reflecting the low uptake amongst responders in the present study.

## Conclusions

Physiotherapists reported adopting a biopsychosocial approach in managing patients with ASI and varied the types of intervention delivered depending upon clinical presentation and psychosocial needs of the patient. Exercise was the most frequently cited intervention, followed by education. The majority of physiotherapists reported either low or moderate confidence in managing ASI with only a small minority report utilising exercise-based management protocols to support and guide their practice. National guidelines are now available to guide physiotherapists in how they manage patients with ASI, and there is a need to share these widely based upon the findings of this study.

A wide range of treatment strategies were reported, however many lacked robust evidence to support their use or clinical effectiveness in ASI. Future research should assess the clinical effectiveness of alternative novel treatment strategies, education and psychologically informed therapies, as well as being directed at facilitating appropriate exercise prescription in this complex patient group.

## Supplementary Information

**Additional file 1. **Atraumatic Shoulder Instability Questionnaire

## Data Availability

The datasets generated and/or analysed during the current study are not publicly available due to the agreed study protocols (as set out in the Participant Information Sheet) that the anonymised data would be stored securely for 3 years following any publication and then destroyed in accordance with the University of Hertfordshire data protection policy (2016) and the Data Protection Act (1998). However, the data is available from the corresponding author on reasonable request.

## Abbreviations

ASI: Atraumatic Shoulder Instability
BESS: British Elbow & Shoulder Society
CSP: Chartered Society of Physiotherapy
DSIRP: Derby Shoulder Instability Rehabilitation Programme
FES: Functional Electrical Stimulation
GMI: Graded Motor Imagery
HCPC: Health & Care Professions Council
MACP: Musculoskeletal Association of Chartered Physiotherapists
NHS: National Health Service
PIT: Psychologically Informed Therapies
QCA: Quantitative Content Analysis
S.D.: Standard Deviation
UK: United Kingdom
