# Supplementary material for: Management of atraumatic shoulder instability in physiotherapy (MASIP): a survey of physiotherapy practice
Source: BMC Musculoskelet Disord. 2021 Sep 30;22:840. doi: 10.1186/s12891-021-04677-9 (PMC8485495; doi:10.1186/s12891-021-04677-9)
Supplement: Supplementary file 1 — Additional file 1. Atraumatic Shoulder Instability Questionnaire [file 12891_2021_4677_MOESM1_ESM.docx]

**Title Page – Appendix**

**Title**

Management of Atraumatic Shoulder Instability in Physiotherapy (MASIP): A survey of physiotherapy practice

**Authors**

Caroline Coulthard, Surrey iMSK Service, Ashford and St. Peter’s Hospitals NHS Foundation Trust, Guildford Road, Chertsey, Surrey, KT16 0PZ United Kingdom

Dr Mindy C Cairns, School of Health and Social Work, University of Hertfordshire, Hatfield, Hertfordshire, AL10 9AB United Kingdom

Deborah Williams, The Shoulder & Elbow Unit, Royal National Orthopaedic Hospital, Brockley Hill, Stanmore, HA7 4LP United Kingdom

Ben Hughes, The Shoulder & Elbow Unit, Royal National Orthopaedic Hospital, Brockley Hill, Stanmore, HA7 4LP United Kingdom

Anju Jaggi, The Shoulder & Elbow Unit, Royal National Orthopaedic Hospital, Brockley Hill, Stanmore, HA7 4LP United Kingdom

**Appendix A - Atraumatic Shoulder Instability Questionnaire**

***Screening Question***

Are you a fully qualified, HCPC registered chartered physiotherapist currently working in the United Kingdom? (Options: Yes, No) *(closed)*

***Demographics***

How many years have you practised as a physiotherapist? (Options: Drop down year selection) *(closed)*

What option most accurately reflects your current professional NHS role or private/educational sector equivalent? (Options: Boxes to select NHS banding, including ‘other’) *(closed)*

In which setting do you work? (Options: National Health Service (NHS), Private hospital, Private physiotherapy practice, Physiotherapy Education, Sports - professional / elite, Sports – semi-professional / amateur, Other) *(closed)*

- *If selected NHS*: Please specify the type of setting? (Options: Primary, Secondary, Tertiary) *(closed)*
- How do clients access your services? (Options: Consultant referral, General Practitioner (GP) referral, Allied Health Professional (AHP) referral, patient self-referral, other - please provide details) *(closed)*

How much overall experience do you have working with clients with musculoskeletal problems? (e.g. total time on rotation in Musculoskeletal +/- time being static if appropriate) (Options: 0-2 yrs, >2-4yrs, >4-6 yrs, >6-8 yrs, >8-10yrs, >10yrs) *(closed)*

***Demographics (2)***

Have you completed any specific training on atraumatic shoulder instability? (Multiple choice) (Options: no specific training completed, post-graduate training, external course, evening lecture, podcast, in-house group training, 1:1 supervision session, online training, ? read journal article or similar, other) *(closed)*

Are you a member of a specialist upper limb clinical interest group or organisation? (Options: Yes, No) *(closed)*

Do you have a clinical or special interest in atraumatic shoulder instability? (Options: Yes, No) *(closed)*

Do you work within a specialist centre for managing patients with shoulder instability? (Options: Yes, No) *(closed)*

Estimate what percentage of your average annual musculoskeletal caseload is made up of patients with atraumatic shoulder instability. (Options: None, >0-10%, >10-30%, >30-50%, >50-75% , >75%) *(closed)*

Do you have any personal experience of atraumatic shoulder instability outside of your workplace setting? (Options: Yes, No, Prefer not to say) *(closed)*

*If yes, second part to question appears:*

a) If yes, please provide details *(open)*

**Vignette 1**

Please read the case scenario below and answer the questions that follow.

26 year old male with atraumatic shoulder instability. Works as an accountant and used to play county cricket but stopped due to developing difficulty with throwing. He has avoided excessive overhead use since then. Complains of sensation of instability and discomfort in right shoulder, particularly on reaching overhead and gardening; been going on for a few years.

He had a Magnetic Resonance Arthrogram (MRA) which shows Bankart lesion of the anterior labrum. He has been referred to physiotherapy by an Extended Scope Practitioner. He now wants to get back to fitness and has started running and upper body weight training. He is struggling with weights due to pain. He wonders whether exercise is the right thing for his shoulder.

On examination he has full range of movement, with notably excessive external rotation. He has a positive gleno-humeral internal rotation deficit (GIRD) test. He has positive anterior apprehension test.

1. Would you use a classification system to help direct your management of this patient?

(Options: Yes, No, Don’t Know) *(closed)*

*If Yes: second & third parts to question appear:*

1. What classification system would you use? *(open)*
2. Using the classification system you have selected, what classification would you give to this patient? *(open)*
3. How would you manage this patient, both initially and as you progress their treatment (please include as much detail as possible)? *(open)*
4. Would you use any particular protocol to guide your management? (Options: Yes, No, Don’t Know) *(closed)*

*If yes, second part to question appears:*

1. If yes, what protocol would you use? *(open)*
2. If the patient doesn't respond to the management you have described, would you consider any of the following options? (please check box and provide details and justify your answer) *(mixed)*
   1. *Different or alternative physiotherapy approaches than any previously described*
   2. *Further investigations*
   3. *Referral to an alternative healthcare professional*
   4. *Other (please state in 'further information' box)*
   5. *None of the above*

**Vignette 2**

24 year old female with bilateral shoulder pain, sensation of instability and history of multiple atraumatic shoulder dislocations. Sometimes attends A&E for help with relocation but normally able to self-relocate, but shoulder will often pop out again shortly after. Owns a dog but struggles to control it on-lead due to feeling shoulder will come out. Unable to work for the past 6 months due to symptoms. Started volunteering at a dog rescue centre but can’t walk the dogs due to shoulder.

Has had episodes of physiotherapy previously, including hydrotherapy and strengthening – feels this hasn't helped overall. Seen a consultant and told not a surgical candidate as no structural pathology on imaging, and referred back for another go at physio. Patient is worried that it won’t help again.

1. Would you use a classification system to help direct your management of this patient?

(Options: Yes, No, Don’t Know) *(closed)*

*If Yes: second part to question appear:*

1. What classification system would you use? *(open)*
2. Using the classification system you have selected, what classification would you give to this patient? *(open)*
3. How would you manage this patient, both initially and as you progress their treatment (please include as much detail as possible)? *(open)*
4. Would you use any particular protocol to guide your management? (Options: Yes, No, Don’t Know) *(closed)*

*If yes, second part to question appears:*

1. If yes, what protocol would you use? *(open)*
2. If the patient doesn't respond to the management you have described, would you consider any of the following options? (please check box and provide details and justify your answer) *(mixed)*
   1. *Different or alternative physiotherapy approaches than any previously described*
   2. *Further investigations*
   3. *Referral to an alternative healthcare professional*
   4. *Other (please state in 'further information' box)*
   5. *None of the above*

**Vignette 3**

26 year old female office worker. History of shoulder bilateral atraumatic shoulder instability with multiple subluxations. Has good social support but lives a relatively sedentary lifestyle. Told was hypermobile when she was younger. No past medical history. Avoids lifting and reaching overhead due to feeling that shoulders will come out.

On examination she has reduced active range of shoulder elevation due to pain, and reports feeling unstable. Range of movement is full passively, with 90 degrees external rotation. Her Beighton score is 7/9. She has a positive sulcus sign, anterior and posterior load and shift, and anterior apprehension and relocation test.

1. Would you use a classification system to help direct your management of this patient?

(Options: Yes, No, Don’t Know) *(closed)*

*If Yes: second part to question appear:*

1. What classification system would you use? *(open)*
2. Using the classification system you have selected, what classification would you give to this patient? *(open)*
3. How would you manage this patient, both initially and as you progress their treatment (please include as much detail as possible)? *(open)*
4. Would you use any particular protocol to guide your management? (Options: Yes, No, Don’t Know) *(closed)*

*If yes, second part to question appears:*

1. If yes, what protocol would you use? *(open)*
2. If the patient doesn't respond to the management you have described, would you consider any of the following options? (please check box and provide details and justify your answer) *(mixed)*
   1. *Different or alternative physiotherapy approaches than any previously described*
   2. *Further investigations*
   3. *Referral to an alternative healthcare professional*
   4. *Other (please state in 'further information' box)*
   5. *None of the above*

***Management Strategies***

How confident are you in managing patients with atraumatic shoulder instability? (Options: Very confident, Somewhat confident, Not confident) *(closed)*

Please rate your awareness, and the extent of your usage, of the following management techniques for patients with atraumatic shoulder instability. (2 parallel Likert scales)

(Likert Scale – Level of Awareness: 1 – Not at all aware, 2 – Slightly aware, 3 – Somewhat aware, 4 – Moderately aware, 5 – Extremely aware) *(closed)*

(Likert Scale – Extent of Usage: 1 – Never, 2 – Seldom, 3 – About half the time, 4 – Usually, 5 – Always) *(closed)*

Topics that will be listed:

- Rotator cuff strengthening exercises
- Postural exercises
- Closed kinetic chain exercise
- Open kinetic chain exercise
- Integrated upper limb & balance exercise
- Scapula stabiliser muscle strengthening
- Therapeutic ultrasound
- Transcutaneous Electrical Nerve Stimulation (TENS)
- Interferential therapy
- Tactile discrimination training
- Laterality training
- Pain management education
- Proprioceptive taping
- Compression garments
- Hydrotherapy
- Massage
- Proprioceptive Neuromuscular Facilitation (PNF)
- Education to enable self-management of atraumatic dislocation
- Explanation of atraumatic shoulder instability to patient
- Joint Position Sense (JPS) training (e.g. target practice or position-matching)
- Activity modification
- Flexibility exercises
- Exercises within ‘safe zone’
- Biofeedback
- Acupuncture
- Functional Electrical Stimulation (FES)

Are there any other strategies that you would consider for managing a patient with atraumatic shoulder instability? (Options: Yes, No) *(closed)*

*If yes, second question is applicable:*

a) If yes, what are these strategies? *(open)*

***Expanding Knowledge of Atraumatic Shoulder Instability***

What, if anything, do you think would improve the ability of physiotherapists to manage patients with atraumatic shoulder instability? (Please tick all that apply and give reasons) (Options: Podcast, In-house training, online course, External course, Evening lecture, Post-graduate training, Working in a specialist setting, Further research, Written guidelines, Other (please state in reasons box)) *(mixed)*

Do you think that further research could help guide your clinical practice with regards to atraumatic shoulder instability? (Options: Yes, No) *(closed)*

*If yes, second question is applicable:*

If yes, what do you think are the key areas to research? *(open)*
